# Another Year

**Published:** 1859-01

**Authors:** 


### EDITORIAL AND MISCELLANEOUS.

### ANOTHER YEAR.

The present number of the *Journal* leaves our sanctum freighted with the kindest regards for our readers, and most ardent wishes that they may each and all experience a happy *New Year.*

Another year has completed its Cycle, and now is numbered with the years before the flood, and though it *is* among "the things that were," yet it, unlike "a schoolboy's tale, the wonder of an hour," has left its mark behind.

Much of the Godless Quackery and Charlatanry, blighting the health and destroying the minds of our fellow creatures, which have disgraced the hours of the old year, have been done in the holy name of science, but 'tis not the fault of the year, and—forgetting what is unpleasant—we will remember the progress of science, and the growth of our race in scientific truth during the same period, and heave a sigh to its memory, and wish that our readers and we, may find the new one as pleasant as the old.

The demand of the age is for Periodical Literature, and each year, new calls are made upon the periodical press, already of gigantic stature and Herculean strength, for light and aid in the discovery of truth and exposition of error. The Medical Press is not exempt from the demand, but the eager cravings of the nineteenth century, clamor loud, and year by year more loudly, upon the Periodical Medical Press, for light in the dark places of the diseases and hidden laws of the human organism.

We are willing to add our labors to those of our cotemporaries, in endeavoring to satisfy this longing; but we are at the sametime admonished, that "all is not gold that glitters," neither is all *good* that is written, or even printed.—We know that in this age, we can have whole pages manufactured of one sentence or a single idea, and may have a fashionable folio furnished us, from which neither reader nor author can gain anything, except it be an exhilarating *gas*, which destroys sense, manners, language, and crazes the mind of the hapless reader. But we are not Homeopathists in Literature any more than in Medical science, therefore, will none of these infinitesimal dilutions for our readers, but having collected our materials, made ready our implements and recuperated our strength, we begin a new year, intending to give them the results of our Medical research, and the facts of the science as they develop themselves, in a concentrated form. In our efforts to make this year a better Journal, than we have ever given to our friends, we ask their aid pecuniarily and promptly—also in extending our circulation, and by their pens—assuring them, we shall be glad to hear from them in either capacity, and promising to do battle for scientific Medicine upon the principles of truth and that reason, which is the strength and dignity of man.

